# Concordance and robustness of quality indicator sets for hospitals: an analysis of routine data

**DOI:** 10.1186/1472-6963-11-106

**Published:** 2011-05-18

**Authors:** Jürgen Stausberg, Axel Halim, Robert Färber

**Affiliations:** 1 Ludwig-Maximilians-Universität München, Institut für Medizinische Informationsverarbeitung, Biometrie und Epidemiologie, München, Germany; 2 Hospital Association of North Rhine-Westphalia, Düsseldorf, Germany

## Abstract

**Background:**

Hospitals are increasingly being evaluated with respect to the quality of provided care. In this setting, several indicator sets compete with one another for the assessment of effectiveness and safety. However, there have been few comparative investigations covering different sets. The objective of this study was to answer three questions: How concordant are different indicator sets on a hospital level? What is the effect of applying different reference values? How stable are the positions of a hospital ranking?

**Methods:**

Routine data were made available to three companies offering the Patient Safety Indicators, an indicator set from the HELIOS Hospital Group, and measurements based on Disease Staging™. Ten hospitals from North Rhine-Westphalia, comprising a total of 151,960 inpatients in 2006, volunteered to participate in this study. The companies provided standard quality reports for the ten hospitals. Composite measures were defined for strengths and weaknesses. In addition to the different indicator sets, different reference values for one set allowed the construction of several comparison groups. Concordance and robustness were analyzed using the non-parametric correlation coefficient and Kendall's W.

**Results:**

Indicator sets differing only in the reference values of the indicators showed significant correlations in most of the pairs with respect to weaknesses (maximum r = 0.927, CI 0.714-0.983, p < 0.001). There were also significant correlations between different sets (maximum r = 0.829, CI 0.417-0.958, p = 0.003) having different indicators or when different methods for performance assessment were applied. The results were weaker measuring hospital strengths (maximum r = 0.669, CI 0.068-0.914, p = 0.034). In a hospital ranking, only two hospitals belonged consistently either to the superior or to the inferior half of the group. Even altering reference values or the supplier for the same indicator set changed the rank for nine out of ten hospitals.

**Conclusions:**

Our results reveal an unsettling lack of concordance in estimates of hospital performance when different quality indicator sets are used. These findings underline the lack of consensus regarding optimal validated measures for judging hospital quality. The indicator sets shared a common definition of quality, independent of their focus on patient safety, mortality, or length of stay. However, for most of the hospitals, changing the indicator set or the reference value resulted in a shift from the superior to the inferior half of the group or vice versa. Thus, while taken together the indicator sets offer the hospitals complementary pictures of their quality, on an individual basis they do not establish a reliable ranking.

## Background

Several quality indicator sets are on the market that measure and assess the quality of hospital acute care provided for inpatients. Quality indicator sets offer several measures (the indicators), with a detailed definition of inclusion and exclusion criteria, formulas for the calculation of each measure, reference values, rules for the frequency of analysis and reporting, as well as recommendations for their appropriate usage. We consider indicator sets not only as a collection of independent measures, but also as a reliable approach to assess quality at the level of the hospital itself or one of its departments, its case groups, managed diseases, or procedures. Individual measures are combined to yield a score or another composite that reflects quality on an aggregate level. The composites might be used, for example, in high-level performance measurements by policy makers and funders.

Literature reviews describe a remarkable number of quality indicator sets, with minor and major overlaps. The Organization for Economic Co-operation and Development (OECD) analyzed six sources to establish their indicators for patient safety at the health systems level [1,2]. The German Advisory Council on the Assessment of Developments in the Health Care System published a report in 2007 on "Cooperation and Responsibility. Prerequisites for Target-Oriented Health Care" [3], with a major focus on quality indicator sets addressing patient safety. The report discusses at least 20 different sets, e.g. the Health Care Quality Indicator set of the OECD, proposals from the US Agency for Healthcare Research and Quality (AHRQ) and from the Joint Commission on Accreditation of Healthcare Organizations, and several national programs, especially in European countries. However, there is little evidence on the effective application of quality indicator sets.

According to Glattacker and Jäckel, the evaluation of quality assurance programs yields results that are "consistently inconsistent," especially concerning the effect on patients' outcome [4]. A recent review of the effective implementation of quality indicators in hospital care identified 21 studies [5]. In most of these, the effects on care processes were evaluated and significant improvements were reported, whereas others determined ineffective results concerning patient outcome. Regarding integrated quality management models, Minkmann et al. concluded, "that there is weak evidence for improved performance" for two out of the three models reviewed in their study [6]. Fung et al. carried out a systematic review of the effect of publicly reported performance data on clinical outcomes and concluded, "The usefulness of public reporting in improving safety and patient-centeredness remains unknown" [7]. It is not surprising, that O'Leary et al. called for the need to "revisit the quality measures currently used for transparency and incentives" [8].

The quality of quality indicators collected in a set is a separate topic. The aim to be RUMBA (cited from [9]), i.e., relevant, understandable, measurable, behavioral, and achievable, is not reached by most of the popular quality indicators, e.g. with respect to hospital mortality [10].

The variety of quality indicator sets, the increasing usage of different sets in Germany, as well as the weak evidence of their usefulness motivated the Hospital Association of North Rhine-Westphalia to establish the "Quality Assessment with Routine Data" project, the aim of which was to obtain a specific quality indicator set based on routine data and targeted for use in German hospitals. Reliable results, no additional workload for data acquisition, and high acceptance by clinicians were the acceptance criteria for the recommended approach. The indicator set should support the hospitals in their negotiations with health insurance funds and other institutions. As part of this project, we analyzed the concordance and robustness of the included quality indicator sets. Specifically, we were interested in answering three questions: (1) How concordant are different indicator sets on a hospital level? (2) What is the effect of applying different reference values? (3) How stable are the positions of a hospital ranking? The answers to these questions are of fundamental importance for the application of quality indicator sets in accreditation, contracting, and public reporting issues.

## Methods

### Project

After a call for participation, three companies signed a contract to participate in the project: 3 M Health Information Systems (abbreviated as 3 M), InMed GmbH (InMed) and Schellen & Partner (Deutschland) GmbH (SP). Ten hospitals in Germany voluntarily provided routine data from 2006. With a mean of 15,196 (standard deviation 7,967), these hospitals serve more inpatients per hospital than German hospitals in general, with a mean of 10,521 inpatients [11].

The routine data included administrative information as well as diagnoses and procedures coded with the legislatively required classifications. The German modification of the International Statistical Classification of Diseases and Related Health Problems 10th Revision (ICD-10-GM) is used for the coding of primary and secondary diagnoses; a national procedure classification is used for the coding of operative and non-operative procedures. Outcome is defined as discharge status living/dead. As the data were anonymized for inpatients as well as for the hospitals, ethical approval of the study was not necessary.

The three companies were expected to deliver standard quality reports within a 3-month period. The deliverables of the companies included the following measures:

• Second-generation quality indicator set of the HELIOS Hospital Group [12], a private operator of more than 60 hospitals in Germany (3 M and InMed); see also http://www.helios-kliniken.de/medizin/qualitaetsmanagement/transparenz/qualitaetskennzahlen.html for information in German.

• Patient Safety Indicators (PSI) of the AHRQ in version 2.1, revision 3 (3 M) [13]; see also http://www.qualityindicators.ahrq.gov/Modules/psi_overview.aspx.

• Analyses based on Disease Staging™ version IV from Medstat Group Inc (SP) [14]. Two manuals are publicly available from the documentation of the US Nationwide Inpatient Sample at http://www.hcup-us.ahrq.gov/db/nation/nis/nisdbdocumentation.jsp.

The second-generation quality indicator set of the HELIOS Hospital Group comprises 78 indicators (e.g. "myocardial infarction mortality rate, age <45") organized in 31 subcategories (e.g. "myocardial infarction") and nine categories (e.g. "diseases of the heart"). Fifty-eight indicators report mortality rates, ten indicators are volume oriented. The PSI count adverse events that could harm the patient. Version 2.1 contains 23 indicators for the evaluation of hospitals. Only two indicators measure mortality. 3 M further divided PSI 4 "failure to rescue" into six particular complications of care, which were also included in this study. Thus, the final PSI set comprised 29 indicators, including eight mortality rates. Disease Staging is a system for the classification of inpatients in disease categories [15]. First, patients are classified in so-called disease categories (DXCat, e.g. "infective endocarditis"). Second, severity of illness is used to define several stages (maximum of five, from normal to death) according to the "risk of organ failure or death." SP uses DXCat and stages as homogeneous groups for an assessment by SP experts. The reports by SP include a sample of DXCat according to the frequency or importance of the disease category.

### Measurements

Composite measures of weaknesses and strengths were calculated for each hospital (cf. method in [16]). To address weaknesses, each indicator result was categorized as inconspicuous or conspicuous by comparing its value to a reference range, provided by either the indicator sets or the companies (yes/no decision). A reference range was established based on a reference value (i.e. a threshold) and a direction for better results. In addition, the number of conspicuous indicator results was divided by the sum of inconspicuous and conspicuous indicator results for each indicator set within each hospital (i.e. relative proportion of conspicuous indicators). Indicators without a reference range were excluded. To address strengths, each indicator result was categorized as best practice or not. An indicator result received the status of best practice if the hospital was the best in class, or among one of the 20% best. In addition, the number of best practice indicator results was divided by the sum of best practice and not best practice indicator results for each indicator set within each hospital (i.e. relative proportion of best practice indicator results).

3 M offered two different reference values for the HELIOS indicator set, the rate calculated from the merged data of all ten hospitals (denoted in this article as "3 M HELIOS group") and a target value published by the HELIOS Hospital Group (3 M HELIOS target) [17]. InMed offered four different references: (1) target value published by the HELIOS Hospital Group (InMed HELIOS target), (2) target value published by the HELIOS Hospital Group compared with confidence limits of the observed rate (InMed HELIOS assessment), (3) a rate calculated from the merged data of a benchmarking data set from the company (InMed HELIOS benchmarking) and (4) current rates published for the HELIOS Hospital Group (InMed HELIOS rates).

InMed defines a confidence interval as follows: The lower limit is defined by the rate calculated by subtracting the number one from the numerator, the upper limit by the rate calculated by adding the number one to the numerator. An observed rate was conspicuous if both lower and upper boundaries were outside the reference range defined by the target value published by the HELIOS Hospital Group (InMed HELIOS assessment).

For PSI, 3 M uses the rate calculated from the merged data of all ten hospitals as reference values (3 M PSI). In contrast to 3 M and InMed, the deliverables of SP included its own assessment of results within DXCat, as established by means of Disease Staging software. The assessment yielded a classification of the length of stay (LOS) distribution using a textual ordinal scale (SP LOS) and a classification of the mortality rate using a color scale (SP Mort): white, yellow and red for inconspicuous, in between and conspicuous, respectively.

Table 1 provides an example of the calculation of the percentage of conspicuous indicators for one hospital. InMed and 3 M calculated the same mortality rate for heart failure in patients age 65-84, i.e. 7.9%. This value was within the confidence limit defined by InMed. Thus, the indicator was rated as inconspicuous in the group "InMed HELIOS assessment." In the case of mortality rates, better quality is indicated by lower values. The observed rate of 7.9% was less than or equal to the reference value of the groups "InMed HELIOS target" (reference value 10.1%), "InMed HELIOS benchmarking" (reference value 9.8%), "3 M HELIOS target" (reference value 10.1%) and "3 M HELIOS group" (reference value 10.5%). Thus, the indicator was rated as inconspicuous for all four groups. However, the observed rate of 7.9% was higher than the reference value of the group "InMed HELIOS rates" (reference value 6.8%). Thus, the indicator was rated as conspicuous for this group. In summary, 8 of 15 indicators of hospital D (53.3%) showed conspicuous results within the group "InMed HELIOS rates." The percentage of 53.3% was then used as the composite measure for weaknesses.

**Table 1:** Example: Observed rates for 15 HELIOS indicators from hospital D

	3 M			InMed				
	Observed rate	Reference value		Observed rate	Confidence limits	Reference value		
Category/subcategory/indicator		Helios target	Helios group			Helios target	Helios rates	Benchmarking
Diseases of the heart: heart attack								
Mortality	7.7%	10.7%	9.5%	7.7%	7.6% - 7.9%	10.7%	8.5%	10.2%
Mortality age <45	0.0%	2.6%	0.9%	0.0%	0.0% - 4.2%	2.6%	1.5%	3.6%
Mortality age 45-64	2.6%	4.3%	3.7%	2.6%	2.1% - 3.1%	4.3%	2.9%	4.2%
Mortality age 65-84	9.9%	12.0%	12.1%	9.9%	9.6% - 10.2%	12.0%	**9.2%**	11.0%
Mortality age >84	15.8%	28.8%	20.8%	15.8%	14.3% - 17.2%	28.8%	23.1%	24.9%
Diseases of the heart: heart failure								
Mortality age >19	9.6%	11.4%	11.4%	9.6%	9.5% - 9.7%	11.4%	**7.9%**	11.0%
Mortality age 20-44	0.0%	4.1%	4.7%	0.0%	0.0% - 7.7%	4.1%	6,0%	4.0%
Mortality age 45-64	0.9%	5.3%	4.4%	0.9%	0.0% - 1.8%	5.3%	2,3%	4.8%
Mortality age 65-84	7.9%	10.1%	10.5%	7.9%	7.7% - 8.1%	10.1%	**6,8%**	9.8%
Mortality age >84	19.7%	18.4%	19.5%	19.7%	**19.3% - 20.1%**	**18.4%**	**17.7%**	**17.8%**
Stroke: all types								
Mortality	19.1%	11.4%	11.8%	19.3%	**18.7% - 19.9%**	**11.4%**	**10.0%**	**10.5%**
Mortality age 20-44	0%	3.8%	2.9%	0.0%	0.0% - 50.0%	3.8%	2.3%	3.4%
Mortality age 45-64	9.1%	5.5%	6.3%	9.1%	0.0% - 16.7%	**5.5%**	**5.4%**	**5.0%**
Mortality age 65-84	16.7%	11.2%	11.5%	16.9%	**15.9% - 17.9%**	**11.2%**	**9.7%**	**10.3%**
Mortality age >84	26.7%	21.0%	24.2%	26.7%	**25.0% - 28.3%**	**21.0%**	**17.6%**	**18.7%**
Percentage conspicuous		33.3%	33.3%		26.7%	33.3%	53.3%	33.3%

Good quality is indicated by low rates for all indicators. Conspicuous results are in bold.

Three different percentages of conspicuous indicators are calculated: 26.7%, 33.3% and 53.3%. Compared with the other hospitals, hospital D received ranks 5, 3, 5, 4, 2 and 2 for the whole HELIOS indicator set.

Overall, each hospital was assigned a maximum of nine composite measures to evaluate its weaknesses, six based on a comparison of its results from the HELIOS indicator set with six different reference values, one based on PSI and two based on Disease Stages. To evaluate its strengths, each hospital was assigned four composite measures, one for the HELIOS indicator set (3 M values: 3 M HELIOS), one for the PSI (3 M values: 3 M PSI), one for the mortality rate of SP within DXCat (SP Mort), and an ordinal assessment by SP of the LOS distribution within DXCat (SP LOS).

Subsequently, the rank of each hospital within each of the 13 comparison groups based on the composite measures was identified, resulting in 90 figures for the assessment of weaknesses, i.e. ten hospitals with the rankings obtained from nine different comparison groups (six HELIOS, PSI, two Disease Staging™), and 40 figures for the assessment of strengths, i.e. ten hospitals with the rankings of four different comparison groups (HELIOS, PSI, two Disease Staging™).

### Statistics

The non-parametric correlation coefficient was calculated according to Spearman in order to assess the concordance of indicator sets. The 95% confidence interval (CI) for the non-parametric correlation coefficient was calculated using the formula presented by Altmann and Gardner [18]. To evaluate the robustness of the indicator sets, the ranks were analyzed using Kendall's coefficient of concordance. Significance was assumed at p ≤ 0.05. The data were managed using a database from Microsoft^® ^Access 2003. Correlation coefficients were calculated with SPSS^® ^14.0, and Kendall's coefficient of concordance with SPSS^® ^15.0.

## Results

Three companies delivered reports for the ten hospitals. Table 2 presents the correlation coefficients for the relative frequency of conspicuous indicator results. There was a significant and substantial correlation between most of the comparison groups covering the HELIOS indicator set, with the highest correlation coefficient (0.927) obtained for the comparison between the 3 M HELIOS and InMed HELIOS targets (CI 0.714-0.983, p < 0.001). The gap to one can be explained by differences either in the indicators calculated, in the indicator results, or in the target values. The coefficients of the other significant correlations from these comparison groups were between 0.657 (CI 0.047-0.910, p = 0.039) and 0.915 (CI 0.673-0.980, p < 0.001). There was no significant correlation between the InMed HELIOS assessment and the two comparison groups defined by 3 M. The use of confidence limits by InMed in this comparison group enhanced the differences between 3 M and InMed in the basic numbers. The correlation between SP Mort and SP LOS was not significant, whereas significant correlations were determined between SP Mort and the InMed HELIOS assessment (r = 0.811, CI 0.371-0.954, p = 0.004), SP Mort and the InMed HELIOS rates (r = 0.723, CI 0.171-0.930, p = 0.018), and SP LOS and 3 M PSI (r = 0.829, CI 0.417-0.958, p = 0.003). Most of the confidence intervals overlapped with each other.

**Table 2:** Correlation coefficients for the relative frequency of conspicuous indicator results

		HELIOS indicator set						3 M PSI	SP Mort	SP LOS
		**3 M HELIOS group**	**3 M HELIOS target**	**InMed HELIOS assessment**	**InMed HELIOS benchmarking**	**InMed HELIOS rates**	**InMed HELIOS target**			

3 M HELIOS group	Coefficient		0.867 (0.523-0.968)	0.571 (-0.092-0.883)	0.903 (0.634-0.977)	0.855 (0.488-0.965)	0.891 (0.595-0.974)	0.304 (-0.403-0.784)	0.413 (-0.293-0.827)	0.426 (-0.278-0.832)
	Sig. (2-sided)		0.001	0.084	0.000	0.002	0.001	0.393	0.235	0.220
3 M HELIOS target	Coefficient			0.559 (-0.109-0.879)	0.915 (0.673-0.980)	0.673 (0.075-0.915)	0.927 (0.714-0.983)	0.000 (-0.630-0.630)	0.292 (-0.414-0.779)	0.085 (-0.575-0.678)
	Sig. (2-sided)			0.093	0.000	0.033	0.000	1.000	0.413	0.815
InMed HELIOS assessment	Coefficient				0.657 (0.047-0.910)	0.802 (0.348-0.951)	0.693 (0.112-0.921)	0.405 (-0.302-0.824)	0.811 (0.371-0.954)	0.409 (-0.253-0.810)
	Sig. (2-sided)				0.039	0.005	0.026	0.245	0.004	0.241
InMed HELIOS benchmarking	Coefficient					0.855 (0.488-0.965)	0.915 (0.673-0.980)	0.036 (-0.607-0.651)	0.529 (-0.151-0.869)	0.188 (-0.501-0.731)
	Sig. (2-sided)					0.002	0.000	0.920	0.116	0.602
InMed HELIOS rates	Coefficient						0.770 (0.272-0.943)	0.353 (-0.356-0.804)	0.723 (0.171-0.930)	0.547 (-0.126-0.875)
	Sig. (2-sided)						0.009	0.318	0.018	0.102
InMed HELIOS target	Coefficient							0.128 (-0.546-0.701)	0.498 (-0.192-0.858)	0.128 (-0.546-0.701)
	Sig. (2-sided)							0.725	0.143	0.725
3 M PSI	Coefficient								0.396 (-0.311-0.821)	0.829 (0.417-0.958)
	Sig. (2-sided)								0.257	0.003
SP Mort	Coefficient									0.341 (-0.368-0.799)
	Sig. (2-sided)									0.334
SP LOS	Coefficient									
	Sig. (2-sided)									

The nine groups consist of 10 hospitals each.

95% confidence interval in parentheses.

**Figure 1:**
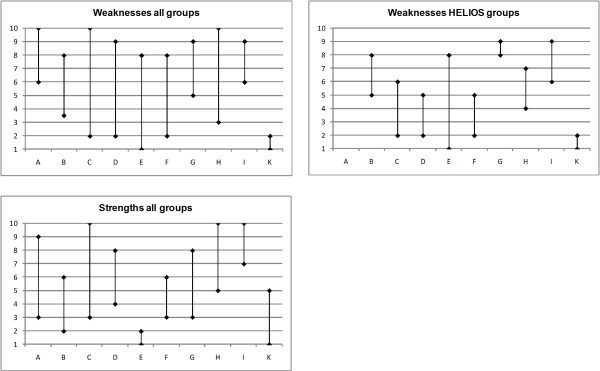
**Range of ranks for weaknesses and strengths**. Hospitals on the horizontal axis, ranks on the vertical axis.

Kendall's coefficient of concordance for all nine groups was 0.598 (p < 0.001), and for the six groups derived from HELIOS indicators 0.825 (p < 0.001). Table 3 and Figure 1 show the ranks of each hospital. According to the HELIOS indicator set, four of the 10 hospitals belonged in some cases to the superior half of the group and in others to the inferior half, depending on the comparison group. Three hospitals belonged consistently to the superior half of the group, and three consistently to the inferior half. Taking into account the results from 3 M PSI, SP Mort and SP LOS as well, only one hospital ranked consistently in the superior group (hospital K) and two in the inferior group (hospitals A and I). The median difference between the lowest and highest rank per hospital was 5.25, with a minimum of 1 and a maximum of 7. The range of the relative frequency of conspicuous indicator results per HELIOS comparison group was at least 31.5% (InMed HELIOS group, minimum 30.8%, maximum 62.3%) and at most 47.7% (InMed HELIOS assessment, 0%, 47.7%). The range was much higher for 3 M PSI, with 68.2% (0%, 68.2%), and for SP LOS, with 85.7% (0%, 85.7%), and much lower for SP Mort, with 13.9% (0%, 13.9%). In the latter case, the low number of conspicuous indicator results, i.e. 26 from 655 (4.0%, 95% confidence interval 2.6%-5.8%), should be noted.

**Table 3:** Rank based on the relative frequency of conspicuous indicator results for each hospital

	HELIOS comparison groups rank			3 M PSI	SP Mort	SP LOS
**Hospital**	**Minimum**	**Maximum**	**Mean**	**Rank**	**Rank**	**Rank**

A	10	10	10.00	6	10	6
B	5	8	6.50	4	5	3.5
C	2	6	3.50	10	6	9
D	2	5	3.50	3	9	3.5
E	1	8	5.17	1	1.5	1
F	2	5	3.33	8	4	5
G	8	9	8.67	5	7	7
H	4	7	5.33	7	3	10
I	6	9	7.83	9	8	8
K	1	2	1.17	2	1.5	2

Rank 1 denotes the best result, and 10 the worst result.

Table 4 presents the correlation coefficients of the relative frequencies of best practice indicator results. There were only two significant correlations, 3 M PSI with SP Mort (r = 0.646, CI 0.028-0.907, p = 0.043) and 3 M PSI with SP LOS (r = 0.669, CI 0.068-0.914, p = 0.034). There was no significant correlation between the two mortality-oriented indicator sets, 3 M HELIOS and SP Mort (r = 0.576, CI -0.084-0.885, p = 0.082). In general, the coefficients were lower than the results of the relative frequencies of conspicuous indicators. All confidence intervals overlapped with each other.

**Table 4:** Correlation coefficients for the relative frequency of best practice indicator results

		3 M HELIOS	3 M PSI	SP Mort	SP LOS
3 M HELIOS	Coefficient		0.372 (-0.336-0.812)	0.576 (-0.084-0.885)	0.310 (-0.397-0.786)
	Sig. (2-sided)		0.290	0.082	0.383
3 M PSI	Coefficient			0.646 (0.028-0.907)	0.669 (0.068-0.914)
	Sig. (2-sided)			0.043	0.034
SP Mort	Coefficient				0.488 (-0.204-0.855)
	Sig. (2-sided)				0.153
SP LOS	Coefficient				
	Sig. (2-sided)				

The four groups consist of 10 hospitals each.

95% confidence interval in parentheses.

Kendall's coefficient of concordance for all four groups was 0.633 (p < 0.001). Only three hospitals (E, I, K) consistently placed in either the superior or inferior half of the group across all four comparison groups (cf. Table 5 and Figure 1). Three hospitals ranked first or second at least once (hospitals B, E and K), whereas four ranked last or second to last at least once (hospitals A, C, H and I). The median difference between the lowest and highest rank per hospital was four, with a minimum of one and a maximum of seven. The range of the relative frequency of best practice indicator results per comparison group was at least 38.9% (3 M HELIOS, minimum 29.9%, maximum 68.8%) and as high as 93.1% (3 M PSI, 6.9%, 100%).

**Table 5:** Rank based on the relative frequency of best practice indicators for each hospital

Hospital	3 M HELIOS	3 M PSI	SP Mort	SP LOS
A	9	5	9	3
B	6	4	4	2
C	3	8	10	7
D	7	7	8	4
E	2	1	1	1
F	4	6	3	6
G	8	3	6	8
H	5	10	5	9
I	10	9	7	10
K	1	2	2	5

Rank 1 denotes the best result, and 10 the worst result.

Comparing the ranks in weaknesses to the ranks in strengths, two hospitals stable ranked in either the superior or the inferior half of the group independent of the comparison group, with hospital K stably positioned in the superior group, and hospital I in the inferior group.

## Discussion

There was substantial correlation between the quality indicator sets analyzed in this study with respect to identification of the weaknesses of the ten hospitals. As expected, indicator sets differing only in their reference values showed significant and substantial correlation coefficients for most of the pairs. Only the results obtained with the proprietary definition of confidence limits by InMed (InMed HELIOS assessment) were not concordant with the results from either 3 M group. The companies' interpretations of the indicator definitions seem to be more relevant than the different ways in which they define the reference values. Therefore, indicator definitions should be made publicly available, non-ambiguous and simple to calculate. Furthermore, arbitrary definitions of established statistical concepts, as is the case with the new definition of confidence limits by InMed, should be firmly rejected.

The mortality analysis using groups defined by Disease Stages showed a strong correlation with two of the indicator sets based on the HELIOS approach. Interestingly, the only significant correlation of PSI was with an analysis based on LOS. One can question whether the adverse events identified by the PSI are more closely related to LOS than to mortality, as represented by the other indicator sets. This might be a "chicken or the egg" type of dilemma since a longer LOS increases the risk of adverse events, including death, but only adverse events other than death increase LOS. The analysis did not identify a favorite candidate for the detection of weaknesses. Instead, the HELIOS indicator set and SP Mort, on the one hand, and the PSI and SP LOS, on the other, might be exchangeable. The few non-overlapping confidence intervals supported the notion of different degrees of concordance between the nine groups of indicator sets.

There was also a substantial correlation between the quality indicator sets identifying the strengths of a hospital. In particular, the correlations between PSI and the assessment of LOS and between PSI and mortality based on Disease Stages were significant. Accordingly, use the PSI may be appropriate if the goal is to identify best practice hospitals. However, the overlapping confidence intervals indicate a large degree of uncertainty in the results. The robustness of the achieved rank is comparable to the results obtained for weaknesses, with a slightly higher Kendall's coefficient of concordance.

The comparison between two hospital ratings, "Hospital Compare" from the US Hospital Quality Alliance and the "Best Hospitals" lists published by U.S. News and World Report, did not result in a demonstration of concordance [19]. The authors of that study calculated a composite score for three conditions as well as a general composite measure. The hospitals covered by "Hospital Compare" were then assigned to quartiles according to the score results. The 14 hospitals named in the Honor Roll of Best Hospitals were equally distributed in the quartiles (5-2-5-2 in 2004, 5-3-6-2 in 2005). Regarding specific conditions, there was a minor correlation between the 50 Best Hospitals and the composite measures of acute myocardial infarction and congestive heart failure, but a minor negative correlation between the 50 Best Hospitals and the composite measure of community-acquired pneumonia. In contrast to the performance measures in "Hospital Compare" and to the indicator sets analyzed in this paper, the Best Hospital rating system covers structural components and a hospital's reputation based on a survey of physicians. Thus, the results presented by Halasyamani and Davis might reflect discrepancies between different players (professionals, health policy, patients), but they do not refute the concordance between the different quality indicator sets examined in our study.

In a simulation study, Jacobs et al. changed the weights used for the construction of a composite measure [20]. The composite measure was based on ten performance indicators covering a broad range of topics, from patient outcome to staffing. Data were obtained from 117 English acute-care hospitals. Unlike our work, Jacobs et al. did not draw any conclusions from a comparison of the results with reference values; they solely concentrated on the relative position with respect to both the value of the composite measure and the rank of the hospital. They concluded that "although the ranking of hospitals at extreme ends of the distribution are reasonably secure - there is considerable uncertainty about the ranking of more central observations," Nevertheless, the correlation coefficients of 0.88 to 0.96 between the original composite measure and four alternatives strengthened their basic concordance.

To some extent, our results explain the conclusions regarding the effectiveness of quality indicators presented in several reviews [4-8]. "Consistently inconsistent" results [4] describe those of low robustness with changing ranks. Nevertheless, the moderate concordance between different sets, each offering its own view of health care services, argues against a radical withdrawal of the recent approaches. The situation is not as bad as claimed by O'Leary et al. [8]. On an aggregate level, adverse events were found to correspond with mortality, and outcome with LOS. Due to the outcome orientation of the analyzed sets, our results do not contribute to a comparison of outcome and process measures.

The use of relative frequency as a composite measure might raise concerns regarding the presented results. There is indeed scant empirical but rather mostly pragmatic evidence for this approach. It does not take into account differences in an indicator's severity, for example. The overlapping confidence intervals in our analyses point out the necessity of a study comprising a larger sample of hospitals. From a statistical point of view, correcting the individual indicator results for small numbers might be preferable. However, the indicator sets use complete data, not samples. The AHRQ itself has discussed composite measures concerning the AHRQ's indicator sets [21,22]. The attempt was made to obtain a single value by combining the particular results of the PSI [22]. Correlation analyses were previously performed for single indicators [23] and for composite measures with different indicator weights [20]. As far as the authors know, the presented work is the first to perform correlation analyses for different indicator sets.

## Conclusions

In accordance with the limited empirical evidence, our results confirm a moderate concordance between different quality indicator sets on an aggregate level and thereby justify the use of these indicator sets for quality management purposes. The information gained from application of the quality indicator sets to routine data can guide hospital management in tailoring quality improvement efforts. However, it is advisable to use more than one set to obtain a complete overview of the provided care, as each set offers a specific view with complementary information on strengths and weaknesses. Nevertheless, we did not analyze the validity or the effectiveness of the sets.

The evaluation of a hospital by a single approach can be very unfair. For the huge group of mean performers, rank depends mainly on the chosen approach, since the quality indicator sets apparently show robust behavior only for the best and worst hospitals. Thus, for a mean performer, a combined assessment using quality indicator sets with financial (e.g. pay for performance) or accreditation (e.g. minimal hospital volume) issues is not supported by our results. Patients must be aware that the current rating systems based on quality indicator sets do not yield a reliable picture of hospital quality. Moreover, hospitals have the opportunity to improve their ranking by selecting a more favorable indicator set of their choice. Much work has to be done to reach an empirically proven application of quality indicator sets that are independent of the support of health professionals.

## Competing interests

The authors declare that they have no competing interests.

## Authors' contributions

AH and RF participated in the design of the study, JS carried out the statistical analysis. JS, AH and RF participated in study coordination. JS drafted the manuscript. All authors read and approved the final manuscript.

## Pre-publication history

The pre-publication history for this paper can be accessed here:

http://www.biomedcentral.com/1472-6963/11/106/prepub
